# Synthesis and Kinase Inhibitory Potencies of Pyrazolo[3,4-*g*]isoquinolines

**DOI:** 10.3390/molecules27175578

**Published:** 2022-08-30

**Authors:** Mathilde Defois, Chloé Rémondin, Béatrice Josselin, Lionel Nauton, Vincent Théry, Fabrice Anizon, Sandrine Ruchaud, Francis Giraud, Pascale Moreau

**Affiliations:** 1 Université Clermont Auvergne, CNRS, Clermont Auvergne INP, ICCF, F-63000 Clermont-Ferrand, France; 2 Sorbonne Université, CNRS, Plateforme de Criblage KISSf (Kinase Inhibitor Specialized Screening Facility), Protein Phosphorylation and Human Diseases Unit, Station Biologique, Place Georges Teissier, F-29688 Roscoff, France; 3 Sorbonne Université/CNRS UMR8227, Station Biologique, Place Georges Teissier, CS90074, CEDEX, F-29688 Roscoff, France

**Keywords:** pyrroloisoquinolines, kinase inhibition

## Abstract

A new series of pyrazolo[3,4-*g*]isoquinoline derivatives, diversely substituted at the 4- or 8-position, were synthesized. The results of the kinase inhibitory potency study demonstrated that the introduction of a bromine atom at the 8-position was detrimental to Haspin inhibition, while the introduction of an alkyl group at the 4-position led to a modification of the kinase inhibition profiles. Altogether, the results obtained demonstrated that new pyrazolo[3,4-*g*]isoquinolines represent a novel family of kinase inhibitors with various selectivity profiles.

## 1. Introduction

**Figure 1:**
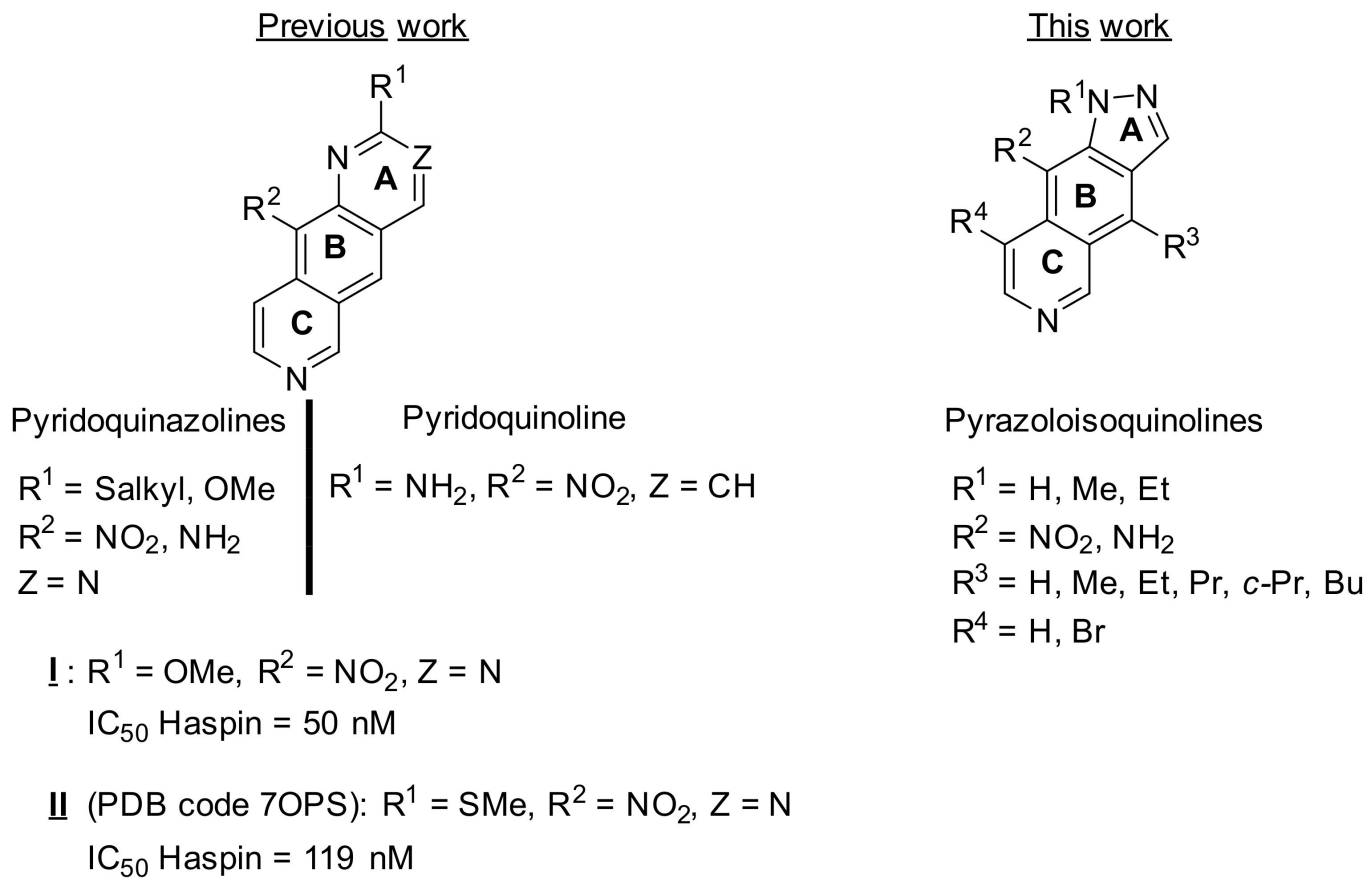
Structure of pyridoquinazolines and pyridoquinoline with Haspin inhibitory potency previously described by our group. Structure of new pyrazolo[3,4-*g*]isoquinolines described in this work.

Protein kinases are implicated in cellular signaling pathways involved in various pathologies such as cancer, neurodegenerative disorders or pain [1]. Therefore, these transferases, able to modulate protein targets by transferring a γ-ATP phosphate group to Ser/Thr and/or Tyr residues, are key targets for identifying new therapeutic strategies. As part of our ongoing studies aiming at identifying new heteroaromatic series with kinase inhibitory potential, we recently described pyridoquinazolines and a pyridoquinoline which were active toward Haspin (haploid germ cell-specific nuclear protein kinase) [2] (Figure 1). Best compounds exhibited nanomolar potencies against Haspin. Haspin is an atypical serine/threonine kinase involved in the phosphorylation of Thr3 (threonine 3) of Histone H3 in mitotic cells. Due to its essential role in mitosis, Haspin appeared as an interesting target for cancer therapy [3]. However, as frequently reported (e.g., [4,5]), Haspin inhibitors cross-inhibited other protein kinases such as CLK1 (cdc2-like kinase 1), DYRK1A (dual-specificity tyrosine-(Y)-phosphorylation regulated kinase 1A) or CDK9 (cyclin-dependent kinase 9). Fortunately, we identified lead compound **I** exhibiting a good activity toward Haspin (IC_50_ Haspin = 50 nM) and selectivity (only 12% of kinase inhibited when tested on a large panel of 486 kinases) (Figure 1). Moreover, we described the molecular interactions established between thio analog **II** and the Haspin ATP binding pocket, showing that inhibitor stabilization mainly involved hydrophobic interactions while a hydrogen bond was established between the pyridine ring and the kinase hinge region residues (PDB code 7OPS).

In this work, to enlarge our structure-activity relationship study and get an insight into the impact of the nature and size of ring **A** on the kinase inhibitory potency of the synthesized compounds, the 6-membered ring was replaced by a pyrazole nucleus (Figure 1).

## 2. Synthesis and Biological Activity of Pyrazolo[3,4-*g*]isoquinolines

**Scheme 1:**
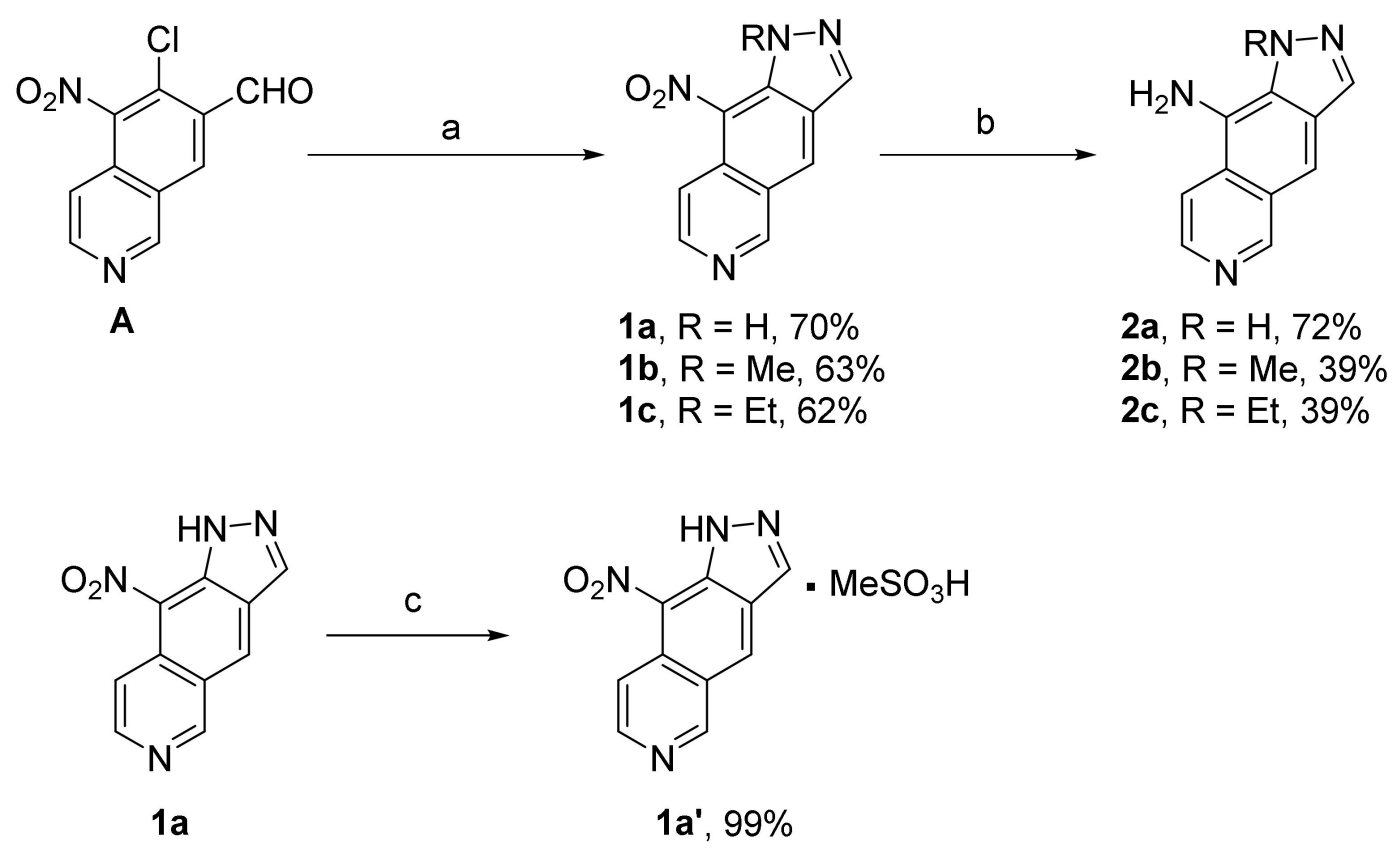
Synthesis of pyrazolo[3,4-*g*]isoquinolines (**1a**–**1c**, **2a**–**2c**) and methanesulfonate salt **1a’**. Reagents and conditions: (**a**) H_2_N-NH_2_.H_2_O or methyl/ethylhydrazinium salts, EtOH (**b**) H_2_, Pd/C, CH_2_Cl_2_/MeOH (**c**) MeSO_3_H, Et_2_O.

In order to modify the size of the upper heterocycle, based on the synthesis of the previous series, we studied the reactivity of intermediate **A** [6,7] with hydrazine or methyl/ethylhydrazinium salts (oxalate or sulfate) (Scheme 1).

In these conditions, pyrazoloisoquinolines **1a**–**1c** were obtained in 62–70% yields. The regiochemistry of the pyrazole ring formation in the presence of alkylhydrazines was attested by 2D NMR experiments. As mentioned below in the biological evaluation part, the very low solubility of compound **1a** led us to prepare the corresponding methanesulfonate salt **1a’**. Amino counterparts **2a**–**2c** were prepared in acceptable to good yields by catalytic hydrogenation of **1a**–**1c** (Scheme 1).

The inhibitory potencies of new compounds **1b**–**1c** and **2a**–**2c** were studied toward a panel of eight protein kinases (Haspin, CLK1, DYRK1A, CDK9/Cyclin T, GSK-3β, CK1ε, CDK5/p25 and Pim1) (Table 1). It should be noted that due to solubility issues, compound **1a** could not be tested; only the corresponding methane sulfonate salt **1a’** was evaluated. The percentage of residual kinase activity was evaluated at 10 μM and 1 μM compound concentrations. Haspin IC_50_ values were determined for compounds with Haspin residual kinase activity <50% at 1 μM compound concentration. In order to assess the selectivity profile of the best inhibitors, IC_50_ values were also measured for other kinases inhibited ≥50% at 1 μM.

Our results indicated that for nitro analogs, the most inhibited kinases were Haspin, CLK1, DYRK1A and CDK9. The most potent Haspin inhibitors were **1b** and **1c** with IC_50_ values of 57 nM and 66 nM and selectivity index (SI) of 1.2 and 2.5, respectively. Best SI in favor of Haspin was observed for less potent inhibitor **1a’**, with Haspin being the sole kinase inhibited to more than 50% at 1 μM compound concentration. Concerning amino analogs, **2c** was the best Haspin inhibitor with an IC_50_ value of 62 nM and a selectivity index of 4 in favor of Haspin versus DYRK1A. Compound **2b**, exhibiting a similar inhibitory profile, was slightly less active toward Haspin. Finally, compound **2a** demonstrated the worst selectivity profile, with six out of eight kinases inhibited to more than 50% at 1 μM. Based on these results, due to better overall selectivity observed in the nitro series, we decided to study the impact of the introduction of various substituent at the 4- or 8-position of this tricyclic scaffold on their kinase inhibitory potency/selectivity. Nitro analog **1c** with the best SI was selected as a starting point.

**Scheme 2:**
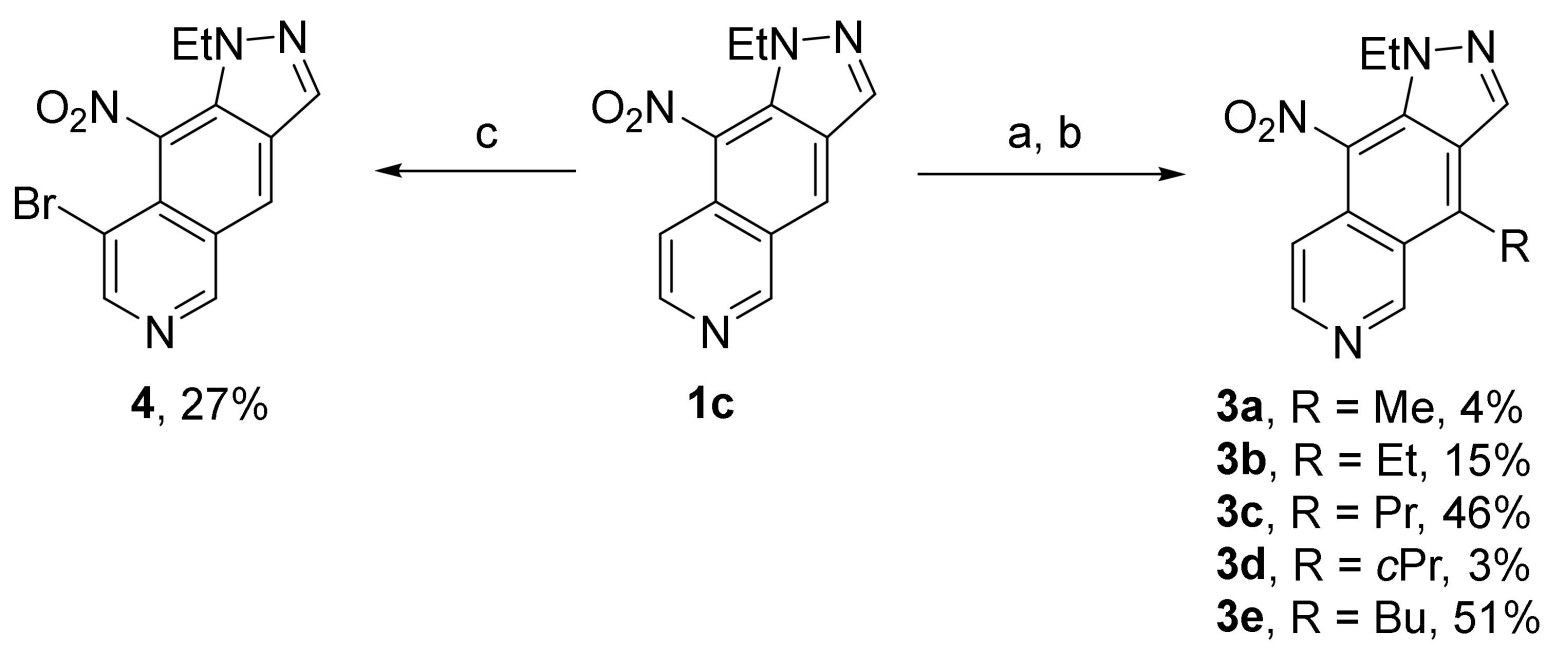
Synthesis of 4-substituted pyrazoloisoquinolines (**3a**–**3e**) and **4**. Reagents and conditions: (**a**) RMgBr, THF (**b**) DDQ, DCM/MeOH 3:2 (**c**) NBS, DMF.

Thus, compound **1c** was reacted in the presence of Grignard reagents before aromatization by DDQ, leading to the corresponding diversely 4-substituted analogs **3a**–**3e**. Compound **1c** was also brominated using NBS in DMF to give **4** in order to study the impact of the substitution of the pyridine part via metallo-catalyzed coupling from a halogenated intermediate (Scheme 2) [8].

The position of the bromine atom was determined by 2D NMR experiments. However, given the low yield of this bromination reaction, this route was not further explored.

The kinase inhibitory potencies of these diversely substituted pyrazoloisoquinolines were studied toward the same panel of protein kinases as above. As indicated in Table 2, the introduction of a bromine atom at the 8-position was detrimental to Haspin inhibition, while the introduction of an alkyl group at the 4-position led to different kinase inhibition profiles. Actually, brominated analog **4** only inhibited Haspin by 23% at 1 μM. Compounds **3b** (4-Et) and **3e** (4-Bu) did not inhibit Haspin to more than 50% at 1 μM. Most potent Haspin inhibitor of this series was **3a** bearing a methyl group (IC_50_ = 167 nM); however, this analog was more potent towards CLK1 (IC_50_ = 101 nM). Compounds **3c** and **3d** bearing a propyl or a cyclopropyl group were more active against CLK1/CDK9/GSK3 (IC_50_ ranging from 218 to 363 nM) compared to Haspin. Altogether these results demonstrated that the alkylation of the 4-position led to a change in the kinase inhibition profile, with CLK1, CDK9 and GSK3 being preferentially inhibited over Haspin.

In order to explain these different results, we undertook molecular modeling experiments to determine the putative binding mode of this new series within the Haspin ATP-binding pocket.

## 3. Molecular Modeling Experiments

Thus, the molecular interactions established between nanomolar inhibitor **1c**, exhibiting a SI in favor of Haspin, as well as those of less potent 4-alkylated analogs **3a** (SI in favor of CLK1) and **3e** (only 22% of Haspin inhibition at 1 μM) were studied by docking. The docking experiments were performed using the Vina-1.2.1 hydrated method [9,10,11]. The Kinase ATP-binding site model was constructed from Protein Data Bank (PDB) 7OPS structure [2]. First of all, in order to validate the molecular modeling protocol used, docking was performed with a 7OPS ligand (**II**, Figure 1). This experiment concluded on the same binding mode as that determined by X-ray crystallography and therefore validated the procedure used.

**Figure 2:**
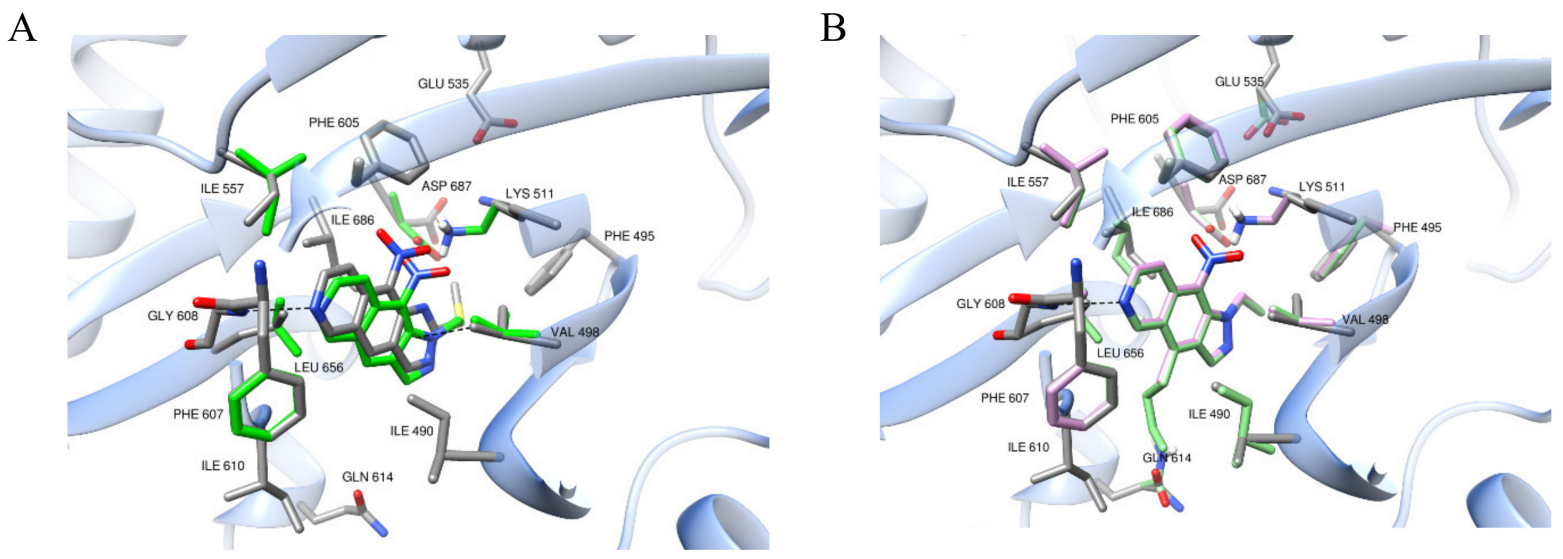
Plausible binding mode of **1c**, **3a** and **3e** within Haspin ATP-binding site. (**A**) Superimposition of **II** (grey color) and **1c** (green color). (**B**) Superimposition of **3a** (purple color) and **3e** (light green) color. The images were produced using UCSF Chimera [12].

As shown in Figure 2A, the tricyclic system of both series is mainly stabilized into the ATP-binding site by hydrophobic interactions. The pyridine moiety is oriented toward the hinge region and establishes an *H*-bond with GLY608 residue (Figure 2A,B). The main difference observed between compound **II** (with an upper pyrimidine ring), and this new series is the offset of the pyrazole ring position with respect to the VAL498 residue. Regarding 4-substituted derivatives (**3a** and **3e**), the heteroaromatic scaffold adopted a similar pose (Figure 2B). Alkyl groups (Me for **3a**, Bu for **3e**) are located in a highly hydrophobic pocket constituted by ILE490, ILE610 and PHE607 residues. However, these interactions failed to improve Haspin inhibitory potency compared to pyrazole derivatives not alkylated at the 4-position.

## 4. Conclusions

In summary, we synthesized a series of pyrazolo[3,4-g]isoquinoline derivatives diversely substituted at the 4- or 8-position. The results of the kinase inhibitory potency study demonstrated that better overall selectivity in favor of Haspin was observed in the nitro series for compounds **1b** and **1c**, with IC_50_ values of 57 nM and 66 nM, respectively. On the other hand, the introduction of a bromine atom at the 8-position was detrimental to Haspin inhibition, while the introduction of an alkyl group at the 4-position led to different kinase inhibition profiles. Finally, docking experiments provided a putative binding mode for this new pyrazolo[3,4-*g*]isoquinoline series within the ATP-binding site. Altogether, the results obtained demonstrated that new pyrazolo[3,4-*g*]isoquinolines represent a novel family of kinase inhibitors with various selectivity profiles.

## 5. Materials and Methods

### 5.1. Chemistry

#### 5.1.1. General

Starting materials were obtained from commercial suppliers and used without further purification. IR spectra were recorded on a Perkin-Elmer Spectrum 65 or Smart Orbit, Nicolet 5700 thermo electron FT-IR spectrometer ($\bar{v}$ in cm^−1^). NMR spectra, performed on a Bruker AVANCE 400 III HD (^1^H: 400 MHz, ^13^C: 101 MHz) or a Bruker AVANCE III HD 500 (^1^H: 500 MHz, ^13^C: 126 MHz), are reported in ppm using the solvent residual peak as an internal standard; the following abbreviations are used: singlet (s), doublet (d), triplet (t), quadruplet (q), quintuplet (quint), hexuplet (hex), heptuplet (hept), multiplet (m) and broad signal (br s). Coupling constants are expressed in Hertz. High resolution mass spectra were determined on a high-resolution Waters Micro Q-Tof or Thermo Scientific Q Exactive Q-Orbitrap apparatus (UCA START, Université Clermont Auvergne, Clermont-Ferrand, France). Chromatographic purifications were performed by column chromatography using 40–63 μm silica gel or by preparative TLC using silica gel-coated glass plates 60 F254 from Macherey Nagel. Reactions were monitored by TLC using fluorescent silica gel plates (60 F254 from Macherey Nagel). Melting points were measured on a Stuart SMP3 apparatus and were uncorrected.

The purity of all tested compounds was established by HPLC analysis using either a VWR Hitachi chromatograph (for **1a**–**1c**, **1a’**, **2a**–**2c**, **3e**, **4**) or an Agilent 1100 series G1315A (for **3a**–**3d**) with DAD detector. A Macherey Nagel Nucleodur gravity column (4.6 mm × 250 mm, 5 µM) was used for all compounds. The flow rate was 0.5 mL/min, and the analysis was performed at 25 °C at 240 or 270 nm as the detection wavelength for each compound. Solvents were (A) water/0.1% formic acid, (B) Acetonitrile. Two methods were designed: method A was a gradient of 5:95 A/B for 5 min to 95:5 A/B in 25 min, whereas method B was an isocratic mode using 60/40 water/acetonitrile.

#### 5.1.2. 9-Nitro-1H-pyrazolo[3,4-*g*]isoquinoline (**1a**)

To a solution of 6-chloro-5-nitroisoquinoline-7-carbaldehyde **A** (100 mg, 0.423 mmol) in EtOH (2.4 mL) was added a solution of hydrazine hydrate 98% (25.3 µL, 0.519 mmol, 1.2 eq.). The reaction mixture was stirred 1 h at room temperature before refluxing for 2 h. After filtration, washing with EtOH and a mixture CH_2_Cl_2_/Acetone (9:1, 50 mL), compound **1a** was obtained as a yellow solid (64 mg, 0.299 mmol, 70%). *R*_f_ = 0.1 (CH_2_Cl_2_/Acetone 9:1). Mp > 250 °C. IR (ATR): 3225-1988, 1624, 1602, 1524, 1455, 1368 cm^−1^. ^1^H NMR (400 MHz, DMSO-*d*_6_) δ 8.77 (1H, d, *J* = 6.4 Hz), 8.87 (1H, s), 8.97 (1H, d, *J* = 6.4 Hz), 9.36 (1H, s), 9.68 (1H, s), 14.18 (1H, br s, NH). ^13^C NMR (100 MHz, DMSO-*d*_6_) δ 115.2, 133.9, 138.6, 147.5, 155.9 (CH_arom_), 99.6, 127.6, 128.1, 135.6, 160.9 (C_arom_). HRMS (ESI+) calcd for C_10_H_7_N_4_O_2_ (M + H)^+^ 215.0563, found 215.0556. HPLC: purity > 95%, λ = 240 nm, t_R_ = 18.6 min (Method B).

#### 5.1.3. 9-Nitro-1H-pyrazolo[3,4-*g*]isoquinoline Methanesulfonate (**1a’**)

To a solution of **1a** (34.2 mg, 0.159 mmol), in anhydrous diethyl ether (10 mL), was slowly added methanesulfonic acid (10 µL, 0.154 mmol, 1.1 eq.). After 10 min of stirring, the brown precipitate formed was filtered off. After trituration with pentane and washing with diethyl ether, **1a’** was obtained as a brown solid (49.3 mg, 0.159 mmol, 99%). Mp > 250 °C. IR (ATR): 3300-2944, 1627, 1529, 1338, 1275 cm^−1^. ^1^H NMR (400 MHz, DMSO-*d*_6_) δ 2.32 (3H, s), 8.81 (1H, d, *J* = 5.2 Hz), 8.96 (1H, s), 9.12 (1H, d, *J* = 5.2 Hz), 9.48 (1H, s), 9.89 (1H, s), 14.37 (1H, br s, NH). ^13^C NMR (100 MHz, DMSO-*d*_6_) δ 117.4, 135.4, 139.2, 140.7, 153.3 (CH_arom_), 122.5, 128.9, 129.0, 136.4, 147.8 (C_arom_). CH_3_ signal under solvent. HRMS (ESI+) calcd for C_10_H_7_N_4_O_2_ (M + H)^+^ 215.0563, found 215.0561. HPLC: purity > 97%, λ = 240 nm, t_R_ = 18.6 min (Method A).

#### 5.1.4. 1-Methyl-9-nitro-1H-pyrazolo[3,4-*g*]isoquinoline (**1b**)

As described for **1a**, using intermediate **A** (70 mg, 0.296 mmol), EtOH (2 mL), methylhydrazine sulfate (51.3 mg, 0.356 mmol, 1.2 eq.), compound **1b** was obtained as a brown solid (42.8 mg, 0.187 mmol, 63%). *R*_f_ = 0.4 (EtOAc). Mp: 202–203 °C. IR (ATR): 1619, 1534, 1458, 1345, 1137 cm^−1^. ^1^H NMR (400 MHz, DMSO-*d*_6_) δ 4.09 (3H, s), 8.21 (1H, d, *J* = 6.8 Hz), 8.67 (1H, d, *J* = 6.8 Hz), 8.97 (1H, s), 9.38 (1H, s), 9.92 (1H, s). ^13^C NMR (100 MHz, DMSO-*d*_6_) δ 38.6 (CH_3_), 115.6, 131.5, 137.3, 139.0, 153.2 (CH_arom_), 121.5, 126.4, 126.6, 129.5, 132.2 (C_arom_). HRMS (ESI+) calcd for C_11_H_9_N_4_O_2_ (M + H)^+^ 229.0720, found 229.0717. HPLC: purity > 95%, λ = 240 nm, t_R_ = 20.8 min (Method A).

#### 5.1.5. 1-Ethyl-9-nitro-1H-pyrazolo[3,4-*g*]isoquinoline (**1c**)

As described for **1a**, using intermediate **A** (500 mg, 2.113 mmol), EtOH (34 mL), ethylhydrazine oxalate (381 mg, 2.536 mmol, 1.2 eq.), the reaction mixture was refluxed for 6 h. The solvent was removed under reduced pressure, and the residue was partitioned between water and ethyl acetate. The organic phase was dried over MgSO_4_ and evaporated. Purification by flash chromatography using CH_2_Cl_2_/Acetone 9:1 as eluent afforded compound **1c** as a brown solid (320.7 mg, 1.325 mmol, 62%). *R*_f_ = 0.55 (CH_2_Cl_2_ /Acetone 9:1). Mp: 123–124 °C. IR (ATR): 1625, 1510, 1341, 1291, 1213 cm^−1^. ^1^H NMR (400 MHz, CD_3_OD) δ 1.43 (3H, t, *J* = 7.2 Hz), 4.48 (2H, q, *J* = 7.2 Hz), 7.94 (1H, d, *J* = 6.4 Hz), 8.54 (1H, d, *J* = 6.4 Hz), 8.66 (1H, s), 9.05 (1H, s), 9.52 (1H, s). ^13^C NMR (100 MHz, CD_3_OD) δ 15.3 (CH_3_), 47.5 (CH_2_), 115.1, 129.3, 137.6, 144.9, 156.0 (CH_arom_), 119.5, 124.3, 127.6, 130.4, 131.7(C_arom_). HRMS (ESI+) calcd for C_12_H_11_N_4_O_2_ (M + H)^+^ 243.0876, found 243.0875. HPLC: purity > 95%, λ = 240 nm, t_R_ = 22.1 min (Method A).

#### 5.1.6. 1-H-pyrazolo[3,4-*g*]isoquinolin-9-amine (**2a**)

To a solution of **1a** (50 mg, 0.233 mmol) in a mixture of CH_2_Cl_2_/MeOH (1:1) (20 mL) was added Pd/C (10 wt%, 9 mg, 0.084 mmol). The reaction mixture was then stirred under H_2_ atmosphere (1 bar) at room temperature for 2 h 30. After filtration over Celite, washing with EtOAc and solvent evaporation, **2a** was obtained as a yellow powder (31 mg, 0.168 mmol, 72%). *R*_f_ = 0.4 (EtOAc/MeOH 9:1). Mp > 250 °C. IR (ATR): 3407-2386, 1645, 1599, 1489, 1366 cm^−1^. ^1^H NMR (400 MHz, DMSO-*d*_6_) δ 6.21 (2H, br s, NH_2_), 7.80 (1H, s), 7.97 (1H, d, *J* = 6.0 Hz), 8.13 (1H, d, *J* = 6.4 Hz), 8.33 (1H, s), 9.23 (1H, s), 12.82 (1H, br s, NH); ^13^C NMR (100 MHz, DMSO-*d*_6_) δ 105.8, 115.0, 134.8, 137.3, 154.7 (CH_arom_), 117.3, 124.7, 125.5, 126.2, 128.9 (C_arom_). HRMS (ESI+) calcd for C_10_H_9_N_4_ (M + H)^+^ 185.0827, found 185.0824. HPLC: purity > 95%, λ = 270 nm, t_R_ = 18.5 min (Method B).

#### 5.1.7. 1-Methyl-1H-pyrazolo[3,4-*g*]isoquinolin-9-amine (**2b**)

As described for **2a**, using **1b** (70 mg, 0.306 mmol), CH_2_Cl_2_/MeOH (1:1) (20 mL), Pd/C (10 wt%, 14 mg, 0.131 mmol), the reaction mixture, protected from light, was stirred under H_2_ atmosphere (1 bar) at room temperature for 1 h 30. After filtration over Celite and solvent evaporation, the crude mixture was purified by flash chromatography using CH_2_Cl_2_/NH_3_ (7N methanolic solution) 98:2 to give **2b** as a brown solid (24 mg, 0.121 mmol, 39%). *R*_f_ = 0.7 (EtOAc/MeOH 9:1). Mp: 174–175 °C. IR (ATR): 3354-2854, 1654, 1584, 1453, 1387, 1182 cm^−1^. ^1^H NMR (400 MHz, DMSO-*d*_6_) δ 4.42 (3H, s), 6.00 (2H, br s, NH_2_), 7.83 (1H, s), 8.10 (1H, d, *J* = 6.4 Hz), 8.15 (1H, d, *J* = 6.4 Hz), 8.24 (1H, s), 9.21 (1H, s). ^13^C NMR (100 MHz, DMSO-*d*_6_) δ 39.1 (CH_3_), 107.5, 114.9, 133.2, 137.9, 154.6 (CH_arom_), 119.2, 125.3, 126.7, 127.2, 129.2 (C_arom_). HRMS (ESI+) calcd for C_11_H_11_N_4_ (M + H)^+^ 199.0978, found 199.0976. HPLC: purity > 95%, λ = 270 nm, t_R_ = 19.1 min (Method A).

#### 5.1.8. 1-Ethyl-1H-pyrazolo[3,4-*g*]isoquinolin-9-amine (**2c**)

As described for **2b**, using **1c** (51 mg, 0.211 mmol), CH_2_Cl_2_/MeOH (1:1) (12 mL), Pd/C (8 mg, 0.075 mmol). Flash chromatography using EtOAc to give **2c** as a yellow gum (17.7 mg, 0.083 mmol, 39%). *R*_f_ = 0,4 (AcOEt). IR (ATR): 3411-2851, 1647, 1571, 1484, 1380 cm^−1^. ^1^H NMR (400 MHz, DMSO-*d*_6_) δ 1.32 (3H, t, *J* = 7.2 Hz), 4.80 (2H, q, *J* = 7.2 Hz), 5.97 (2H, br s, NH_2_), 7.85 (1H, s), 8.13 (1H, d, *J* = 6.4 Hz), 8.16 (1H, d, *J* = 6.4 Hz), 8.29 (1H, s), 9.22 (1H, s). ^13^C NMR (100 MHz, DMSO-*d*_6_) δ 16.7 (CH_3_), 46.2 (CH_2_), 107.6, 115.0, 134.0, 138.0, 154.5 (CH_arom_), 119.5, 125.3, 126.8, 127.2, 128.4 (C_arom_). HRMS (ESI+) calcd for C_12_H_13_N_4_ (M + H)^+^ 213.1135, found 213.1135. HPLC: purity > 95%, λ = 240 nm, t_R_ = 19.8 min (Method A).

#### 5.1.9. General Procedure for the Alkylation of 1-Ethyl-9-nitro-1H-pyrazolo[3,4-*g*]isoquinoline **1c**

Preparation of Grignard reagents:

Except for methylmagnesium bromide (1.92 M in diethyl ether) and ethylmagnesium bromide (0.33 M in THF) solutions that were purchased from Sigma-Aldrich, all other organomagnesium reagents were prepared according to the following procedure.

Magnesium turnings (100 mg, 4.1 mmol, 1 eq) in 2 mL of anhydrous THF were activated by adding a small amount of iodine. Then the suitable bromide (4.1 mmol, 1 eq) was added dropwise at 0 °C. Once the addition finished, the mixture was heated to reflux for 45 min. The concentration of the obtained organomagnesium solution was determined as follows: to a solution of iodine (100 mg) in anhydrous THF was added dropwise the organomagnesium solution until the complete disappearance of iodine color.

The reaction of organomagnesium/organolithium reagents with compound **1c**:

To a solution of compound **1c** in anhydrous THF (0.05 mmol/mL) was added dropwise a solution of the corresponding organomagnesium derivative. The mixture was stirred at a suitable temperature under an argon atmosphere until all the starting material was consumed (TLC control, from 3 to 18 h). The mixture was then treated with a saturated aqueous NH_4_Cl solution, made alkaline with a saturated aqueous NaHCO_3_ solution and extracted with EtOAc. Combined organic layers were dried over MgSO_4_ and concentrated. The crude material was used in the next step without purification as follows: the residue was solubilized in a mixture of DCM/MeOH (3/2, 0.01 mmol/mL), then DDQ (2,3-dichloro-5,6-dicyano-*p*-benzoquinone) (1 eq) was added. The solution was stirred at room temperature until the TLC control showed that the additional product was totally oxidized (1 h). The reaction mixture was then concentrated under reduced pressure. The residue was purified by flash chromatography or/and preparative TLC to give the corresponding 5-substituted derivative.

#### 5.1.10. 1-Ethyl-4-methyl-9-nitro-1H-pyrazolo[3,4-*g*]isoquinoline (**3a**)

Compound **1c** (50 mg, 0.206 mmol), methylmagnesium bromide (1.92 M/Et_2_O, 0.43 mL, 0.826 mmol), reflux, 12 h. Then DDQ (187 mg, 0.826 mmol) in DCM/MeOH 3:2 mixture (60 mL). Purification by preparative TLC using DCM/Acetone 10% (two runs) to give compound **3a** (2.1 mg, 0.008 mmol, 4%) as a yellow powder. *R*_f_ = 0.45 (DCM/MeOH 9:1). Mp. 92–93 °C. IR (ATR): 2931, 1675, 1506, 1456, 1377, 1340 cm^−1^. ^1^H NMR (400 MHz, DMSO-*d*_6_) δ 1.32 (3H, t, *J* = 7.2 Hz), 3.21 (3H, s), 4.36 (2H, q, *J* = 7.2 Hz), 7.88 (1H, d, *J* = 6.4 Hz), 8.62 (1H, d, *J* = 6.4 Hz), 9.01 (1H, s), 9.77 (1H, s). ^13^C NMR (100 MHz, DMSO-*d*_6_) δ 14.8, 15.2 (CH_3_), 46.1 (CH_2_), 113.1, 136.3, 145.1, 151.8 (CH_arom_), 120.6, 124.8, 126.4, 128.5, 129.7, 139.8 (C_arom_). HRMS (ESI+) calcd for C_13_H_13_N_4_O_2_ (M + H^+^) 257.1033, found 257.1025. HPLC: purity > 96%, λ = 270 nm, t_R_ = 20.2 min (Method A).

#### 5.1.11. 1,4-Diethyl-9-nitro-1H-pyrazolo[3,4-*g*]isoquinoline (**3b**)

Compound **1c** (150 mg, 0.619 mmol), ethylmagnesium bromide (0.33 M/THF, 5.63 mL, 1.858 mmol), −40 °C, 4 h. Then DDQ (141 mg, 0.619 mmol) in DCM/MeOH 3:2 mixture (60 mL). Purification by two flash chromatographies first with EtOAc/cyclohexane 1:1 + 0.5% Et_3_N then with DCM/MeOH 5% to give compound **3b** (25 mg, 0.092 mmol, 15%) as a yellow powder. *R*_f_ = 0.50 (DCM/MeOH 9:1). Mp. 96–97 °C. IR (ATR): 2977, 1602, 1502, 1490, 1375, 1271 cm^−1^. ^1^H NMR (400 MHz, DMSO-*d*_6_) δ 1.33 (3H, t, *J* = 7.2 Hz), 1.40 (3H, t, *J* = 7.6 Hz), 3.72 (2H, q, *J* = 7.6 Hz), 4.36 (2H, q, *J* = 7.2 Hz), 7.89 (1H, d, *J* = 6.4 Hz), 8.62 (1*H*, d, *J* = 6.0 Hz), 9.03 (1*H*, s), 9.82 (1*H*, s). ^13^C NMR (100 MHz, DMSO-*d*_6_) δ 14.8, 16.8 (CH_3_), 22.1, 46.0 (CH_2_), 113.3, 135.8, 145.0, 151.4 (CH_arom_), 119.6, 125.0, 126.6, 127.8, 129.7, 145.6 (C_arom_). HRMS (ESI+) calcd for C_14_H_15_N_4_O_2_ (M + H^+^) 271.1189, found 271.1195. HPLC: purity > 98%, λ = 270 nm, t_R_ = 21.1 min (Method A).

#### 5.1.12. 1-Ethyl-9-nitro-4-propyl-1H-pyrazolo[3,4-*g*]isoquinoline (**3c**)

Compound **1c** (50 mg, 0.206 mmol), propylmagnesium bromide (0.36 M/THF, 1.72 mL, 0.619 mmol), 0 °C, 3 h. Then DDQ (47 mg, 0.206 mmol) in DCM/MeOH 3:2 mixture (20 mL). Purification by two flash chromatographies first with EtOAc/cyclohexane 1:1 + 0.5% Et_3_N then with DCM/Acetone 2% to give compound **3c** (27 mg, 0.095 mmol, 46%) as a yellow powder. *R*_f_ = 0.55 (DCM/Acetone 9:1). Mp. 109–110 °C. IR (ATR): 3119, 1605, 1504, 1450, 1338, 1255 cm^−1^. ^1^H NMR (400 MHz, DMSO-*d*_6_) δ 1.00 (3H, t, *J* = 7.2 Hz), 1.33 (3H, t, *J* = 7.2 Hz), 1.80 (2H, hex, *J* = 7.2 Hz), 3.68 (2H, t, *J* = 7.4 Hz), 4.36 (2H, q, *J* = 7.2 Hz), 7.89 (1H, d, *J* = 6.0 Hz), 8.62 (1H, d, *J* = 6.0 Hz), 9.04 (1H, s), 9.83 (1H, s). ^13^C NMR (100 MHz, DMSO-*d*_6_) δ 14.0, 14.8 (CH_3_), 25.5, 30.3, 46.1 (CH_2_), 113.3, 136.1, 145.0, 151.6 (CH_arom_), 120.1, 125.1, 126.6, 128.4, 129.6, 144.0 (C_arom_). HRMS (ESI+) calcd for C_15_H_17_N_4_O_2_ (M + H^+^) 285.1346, found 285.1339. HPLC: purity > 99%, λ = 270 nm, t_R_ = 22.2 min (Method A).

#### 5.1.13. 4-Cyclopropyl-1-ethyl-9-nitro-1H-pyrazolo[3,4-*g*]isoquinoline (**3d**)

Compound **1c** (100 mg, 0.413 mmol), cyclopropylmagnesium bromide (0.44 M/THF, 2.81 mL, 1.236 mmol), 0 °C, 6 h. Then DDQ (94 mg, 0.413 mmol) in DCM/MeOH 3:2 mixture (40 mL). Purification by three chromatographies first by flash column with DCM/Acetone 10% then by a first preparative TLC eluted with DCM/Acetone 10% then a second one using EtOAc to give compound **3d** (4.3 mg, 0.015 mmol, 3%) as a yellow powder. *R*_f_ = 0.60 (DCM/Acetone 9:1). Mp. 97 °C. IR (ATR): 2927, 1604, 1501, 1450, 1331, 1271 cm^−1^_._ ^1^H NMR (400 MHz, DMSO-*d*_6_) δ 1.03-1.09 (2H, m), 1.33 (3H, t, *J* = 7.2 Hz), 1.43–1.50 (2H, m), 2.78–2.87 (1H, m), 4.36 (2H, q, *J* = 7.2 Hz), 7.87 (1H, d, *J* = 6.4 Hz), 8.64 (1H, d, *J* = 6.0 Hz), 8.94 (1H, s), 10.12 (1H, s). ^13^C NMR (100 MHz, CDCl_3_) δ 11.3 (CH_3_), 7.8 (2CH_2_), 46.7 (CH_2_), 15.2 (CH), 113.9, 135.3, 144.8, 152.3 (CH_arom_), 122.9, 127.1, 128.4, 129.3, 130.4, 142.0 (C_arom_). HRMS (ESI+) calcd for C_15_H_15_N_4_O_2_ (M + H^+^) 283.1189, found 283.1182. HPLC: purity > 98%, λ = 270 nm, t_R_ = 21.3 min (Method A).

#### 5.1.14. 4-Butyl-1-ethyl-9-nitro-1H-pyrazolo[3,4-*g*]isoquinoline (**3e**)

Compound **1c** (100 mg, 0.413 mmol), butylmagnesium bromide (0.17 M/THF, 7.16 mL, 1.239 mmol), 0 °C, 3 h. Then DDQ (94 mg, 0.413 mmol) in DCM/MeOH 3:2 mixture (40 mL). Purification by flash chromatography with DCM/Acetone 3% to give compound **3e** (63 mg, 0.211 mmol, 51%) as a yellow powder. *R*_f_ = 0.60 (DCM/Acetone 9:1). Mp. 120–121 °C. IR (ATR): 2970, 1612, 1506, 1456, 1336, 1300 cm^−1^. ^1^H NMR (400 MHz, DMSO-*d*_6_) δ 0.92 (3H, t, *J* = 7.2 Hz), 1.34 (3H, t, *J* = 7.2 Hz), 1.43 (2H, hex, *J* = 7.2 Hz), 1.75 (2H, quint, *J* = 7.2 Hz), 3.71 (2H, t, *J* = 7.6 Hz), 4.36 (2H, q, *J* = 7.2 Hz), 7.89 (1H, d, *J* = 6.4 Hz), 8.62 (1H, d, *J* = 6.0 Hz), 9.03 (1H, s), 9.82 (1H, s). ^13^C NMR (100 MHz, DMSO-d_6_) δ 13.8, 14.8 (CH_3_), 22.3, 28.4, 34.3, 46.0 (CH_2_), 113.3, 136.0, 144.9, 151.5 (CH_arom_), 120.0, 125.1, 126.6, 128.3, 129.6, 144.2 (C_arom_). HRMS (ESI+) calcd for C_16_H_19_N_4_O_2_ (M + H^+^) 299.1502, found 299.1498. HPLC: purity > 95%, λ = 270 nm, t_R_ = 24.1 min (Method A).

#### 5.1.15. 8-Bromo-1-ethyl-9-nitro-1H-pyrazolo[3,4-*g*]isoquinoline (**4**)

To a solution of compound **1c** (30 mg, 0.124 mmol) in DMF (2 mL) at room temperature under argon was added freshly recrystallized *N*-bromosuccinimide (22 mg, 0.124 mmol). The reaction mixture was stirred overnight at room temperature. Two other portions of NBS (6 mg each, 0.037 mmol) were successively added after 24 and 48 h, but no complete conversion was reached. EtOAc was added, and the organic phase was washed with saturated aqueous Na_2_S_2_O_3_ and NaHCO_3_ solutions, dried over MgSO_4_, filtered and evaporated under reduced pressure. Residue was purified by flash chromatography (EtOAc/cyclohexane 1:1) yielding compound **4** (10.8 mg, 0.034 mmol, 27%) as an orange powder, *R_f_* = 0.35 (EtOAc/cyclohexane 1:1). Mp = 172–173 °C; IR (ATR): 2932, 1623, 1525, 1456, 1286 cm^−^^1^; ^1^H NMR (400 MHz, DMSO-*d*_6_) δ 1.37 (3H, t, *J* = 7.2 Hz), 4.31 (2H, q, *J* = 7.2 Hz), 8.82 (1H, s), 8.91 (1H, s), 9.21 (1H, s), 9.62 (1H, s). ^13^C NMR (100 MHz, DMSO-*d*_6_) δ 15.6 (CH_3_), 44.9 (CH_2_), 127.9, 136.5, 148.3, 155.4 (CH_arom_), 110.3, 121.2, 124.0, 126.2, 128.3, 130.2 (C_arom_). HRMS (ESI+) calcd for C_12_H_10_^79^BrN_4_O_2_ (M + H^+^) 320.9982, found 320.9976. HRMS (ESI+) calcd for C_12_H_10_^81^BrN_4_O_2_ (M + H^+^) 322.9961, found 322.9953. HPLC: purity > 95%, λ = 240 nm, t_R_ = 30.3 min (Method A).

### 5.2. In Vitro Kinase Inhibition Assays

Kinase enzymatic activities were assayed in 384 well plates using the ADP-GloTM assay kit (Promega, Madison, WI, USA) according to the manufacturer’s guidance [13]. Briefly, the reactions were carried out in a final volume of 6 μL for 30 min at 30 °C in an appropriate kinase buffer (10 mM MgCl_2_, 1 mM EGTA, 1 mM DTT, 25 mM Tris-HCl pH 7.5, 50 μg/mL heparin), with either protein or peptide as substrate in the presence of 10 μM ATP (40 mM Tris pH 7.5, 20 mM MgCl_2_ and 0.1 mg/mL of BSA). Afterward, 6 μL of ADP-GloTM Kinase Reagent was added to stop the kinase reaction. After an incubation time of 50 min at room temperature (RT), 12 μL of Kinase Detection Reagent was added for 1 h at RT. The transmitted signal was measured using the Envision (Perkin Elmer, Waltham, MA, USA) microplate luminometer and expressed in relative light unit (RLU). In order to determine the half maximal inhibitory concentration (IC_50_), the assays were performed in duplicate in the absence or presence of increasing doses of the tested compounds. GraphPad Prism6 software (GraphPad Software, San Diego, CA, USA) was used to fit dose–response curves and to determine the IC_50_ values. Kinase activities are expressed in the percentage of maximal activity, i.e., measured in the absence of an inhibitor. Peptide substrates were obtained from Proteogenix (Schiltigheim, France).

The following protein kinases were analyzed in this study: CDK5/p25 (human, recombinant, expressed in bacteria) was assayed with 0.8 μg/μL of histone H1 as substrate; CDK9/CyclinT (human, recombinant, expressed by baculovirus in Sf9 insect cells) were assayed with 0.27 μg/μL of the following peptide: YSPTSPSYSPTSPSYSPTSPSKKKK, as substrate; CK1ε (human, recombinant, expressed by baculovirus in Sf9 insect cells) was assayed with 0.022 μg/μL of the following peptide: RRKHAAIGSpAYSITA (“Sp” stands for phosphorylated serine) as CK1-specific substrate; GSK3β (human, recombinant, expressed by baculovirus in Sf9 insect cells) were assayed with 0.010 μg/μL of GS-1 peptide, a GSK-3-selective substrate (YRRAAVPPSPSLSRHSSPHQSpEDEEE); Haspin-kd (human, kinase domain, amino acids 470–798, recombinant, expressed in bacteria) was assayed with 0.007 μg/μL of histone H3 (1–21) peptide (ARTKQTARKSTGGKAPRKQLA) as substrate; Pim-1 (human proto-oncogene, recombinant, expressed in bacteria) was assayed with 0.8 μg/μL of histone H1 as substrate; MmCLK1 (from *Mus musculus*, recombinant, expressed in bacteria) was assayed with 0.027 μg/μL of the following peptide: GRSRSRSRSRSR as substrate; RnDYRK1A-kd (*Rattus norvegicus*, amino acids 1–499 including the kinase domain, recombinant, expressed in bacteria, DNA vector kindly provided by Dr. W. Becker, Aachen, Germany) was assayed with 0.033 μg/μL of the following peptide: KKISGRLSPIMTEQ as substrate.

### 5.3. Molecular Modeling Experiments

The docking studies were performed with the Vina-1.2.1 hydrated method [9,10,11]. Files for the docking were prepared from the PDB 7OPS Haspin structure [2] after removing water molecules. **1c**, **3a** and **3e** pdbqt files were prepared with AutoDockTools (ADT) [14]. Apolar hydrogen atoms were removed, and AM1 charges were added [12]. The geometry of each compound was optimized using Gaussian software (DFT/B3LYP/6-31G) [15]. Docking experiments were performed in accordance with AutoDock Vina 1.2.1 documentation [10,11].

## Figures and Tables

**Table 1:** Percentage of kinase residual activity at 10 μM and 1 μM compound concentrations (**1a’**, **1b**–**1c**, **2a**–**2c**). IC_50_ values in nM (given in parentheses) were determined for Haspin when residual activity was <50% at 1 μM. For other kinases, IC_50_ values were measured for most inhibited ones (% Inhibition ≥ 50% at 1 μM). For compounds **I**, **II**, **1b**, **1c** and **2a**, Selectivity Index (SI) Haspin vs. CLK1 = CLK1 IC_50_/Haspin IC_50_. For compounds **2b** and **2c**, SI was calculated for Haspin vs. DYRK1A, which was more inhibited than CLK1. For **1a’**, SI was estimated to be >6.5 in regard to Haspin IC_50_ value, and CLK1/DYRK1A IC_50_ values were assumed to be >1 μM on the basis of % of inhibition at 1 μM (less than 50% for CLK1/DYRK1A). Assays were performed in duplicate using the ADP-Glo assay in the presence of 10 μM ATP. Typically, the standard deviation of single data points was <10%. Compounds **I** and **II** IC_50_ values from reference [2].

Cpds	Kinase Inhibition (% Residual Activity at 10 μM or 1 μM (IC_50_ Values))																
	Haspin		CLK1		DYRK1A		CDK9		GSK3		CK1		CDK5		PIM1		SI
	10	1	10	1	10	1	10	1	10	1	10	1	10	1	10	1	
**I**	(50)		(445)		(917)												8.9
**II**	(119)		(221)		(916)												1.9
**1a’**	23	32	30	90	25	68	48	86	50	99	64	100	62	91	85	90	>6.5
	(154)																
**1b**	9	26	10	42	11	41	35	57	33	73	53	88	43	95	54	100	1.2
	(57)		(71)		(681)												
**1c**	15	38	6	25	18	44	23	46	19	53	58	90	79	94	70	100	2.5
	(66)		(166)		(1114)		(428)										
**2a**	8	13	8	20	12	32	10	22	18	48	28	71	17	45	90	100	2.3
	(127)		(296)		(600)		(430)		(411)				(927)				
**2b**	14	10	8	36	5	18	15	39	44	68	42	77	60	72	71	91	2.4
	(112)		(548)		(273)		(578)										
**2c**	3	17	8	34	6	23	50	80	40	82	48	84	28	65	60	91	4.0
	(62)		(564)		(250)												

**Table 2:** Percentage of kinases residual activity at 10 μM and 1 μM compound concentration. IC_50_ values in nM (given in parentheses) were determined for Haspin when residual activity was <50% at 1 μM. For other kinases, IC_50_ values were measured for most inhibited ones (Inhibition % ≥ 50% at 1 μM). Kinase inhibitory activities of **3a**–**3e** and **4** were assayed in duplicate using the ADP-Glo assay in the presence of 10 μM ATP. Typically, the standard deviation of single data points was below 10%.

Cpds	Kinase Inhibition (% Residual Activity at 10 μM or 1 μM (IC_50_ Values))															
	Haspin		CLK1		DYRK1A		CDK9		GSK3		CK1		CDK5		PIM1	
	10	1	10	1	10	1	10	1	10	1	10	1	10	1	10	1
**3a**	8	20	0	13	13	73	14	43	9	39	45	89	35	64	97	100
	(167)		(101)				(789)		(1077)							
**3b**	10	63	0	19	9	100	0	25	3	28	25	82	16	33	89	100
**3c**	17	25	4	23	41	95	4	18	5	12	35	81	10	54	88	86
	(497)		(363)				(322)		(218)							
**3d**	12	21	5	24	29	67	11	33	5	17	15	61	24	67	82	99
	(312)		(229)				(266)		(226)							
**3e**	19	78	16	58	16	72	10	33	10	39	60	100	57	82	35	78
**4**	23	77	18	42	25	52	18	59	80	88	80	100	32	92	54	77

## Data Availability

Data are available under request.
